# A Rare Case of Right Fourth Branchial Fistula: A Diagnostic Challenge

**DOI:** 10.7759/cureus.61905

**Published:** 2024-06-07

**Authors:** Faseeha Nur, Ling Xiu Ngui, Chew Shiun Chuen

**Affiliations:** 1 Ear, Nose, and Throat, Hospital Sibu, Sibu, MYS; 2 Otolaryngology, Hospital Sibu, Sibu, MYS

**Keywords:** recurrent infection, branchial cyst, fistula, branchial anomalies, fourth

## Abstract

Fourth branchial anomalies are extremely rare and are often misdiagnosed. A recurrent history of anterior neck discharges or infections since childhood should raise a high clinical suspicion of branchial fistula and necessitate a thorough clinical, endoscopic, and radiological evaluation. We report a rare case of right-sided fourth branchial fistula in a middle-aged lady who was referred to us for recurrent right neck infections since childhood and had received multiple courses of antibiotics and drainage of abscesses. Despite previous negative barium swallow and fistulogram results, the diagnosis of the branchial fistula was made clinically with the spillage of methylene blue dye into the apex of the right pyriform sinus from flexible nasopharyngolaryngoscopy in the clinic after the injection of dye through the fistula opening at the neck. Finally, another barium swallow study and computed tomography scan were conducted, revealing the fistula tract. Complete surgical excision of the fistula tract was then performed with no evidence of recurrence after six months of follow-up.

## Introduction

Development of head and neck structures is derived from pharyngeal arches (formerly known as branchial arches) which appear during the fourth and fifth week of embryonic life [1]. There are six pairs of branchial arches, with each branchial arch consisting of mesenchymal core tissues derived from mesoderm and neural crest cells that are covered externally by ectoderm and lined internally by endoderm known as branchial clefts and branchial pouches, respectively. Abnormal embryogenesis of branchial apparatus can result in various congenital anomalies which include branchial cysts, sinuses, and fistulas. The most accepted theory in the formation of branchial anomalies is the embryonic remnant resulting from the incomplete obliteration of the branchial apparatus [2].

Branchial cleft anomalies are the second most common head and neck congenital lesions in children, with 95% of the anomalies related to second branchial clefts and the remaining mostly arising from first and third branchial arches [3]. Fourth branchial arch anomalies are indeed rare with approximately 40 cases reported in the literature since 1972 [4].

Most anomalies are present during the first decade of life. Third and fourth branchial anomalies have been reported to be present at any age [5]. Fourth branchial anomalies usually present as recurrent neck infections and/or abscesses or acute suppurative thyroiditis [6,7]. The most predominant lesion is almost always seen over the left side [4,5] and rarely on the right side [8]. Fistula of fourth branchial anomalies have an internal opening in the apex of the pyriform sinus, piercing the larynx near the cricothyroid ligament and then passes between the superior and recurrent laryngeal nerve with the external opening at the neck on the lower third anterior border of the sternocleidomastoid muscle.

## Case presentation

A 46-year-old lady was referred to us with a history of on and off right anterior neck swelling with discharges and recurrent infections since childhood. She had no dysphagia, odynophagia, voice changes, dyspnea, or any ear symptoms. There was no history of trauma, insect bites, constitutional symptoms, or any tuberculosis contacts.

For the most recent episode of infection, she visited a private hospital and was treated for a right neck abscess which resolved after a course of oral antibiotics. However, intermittent neck discharges persisted. The barium swallow study was unremarkable. The fistulogram only detected a short sinus track of 5-6 mm from the skin opening seen at the anterior lower neck, and no fistula connection was detected. She was then referred to us for further management.

**Figure 1:**
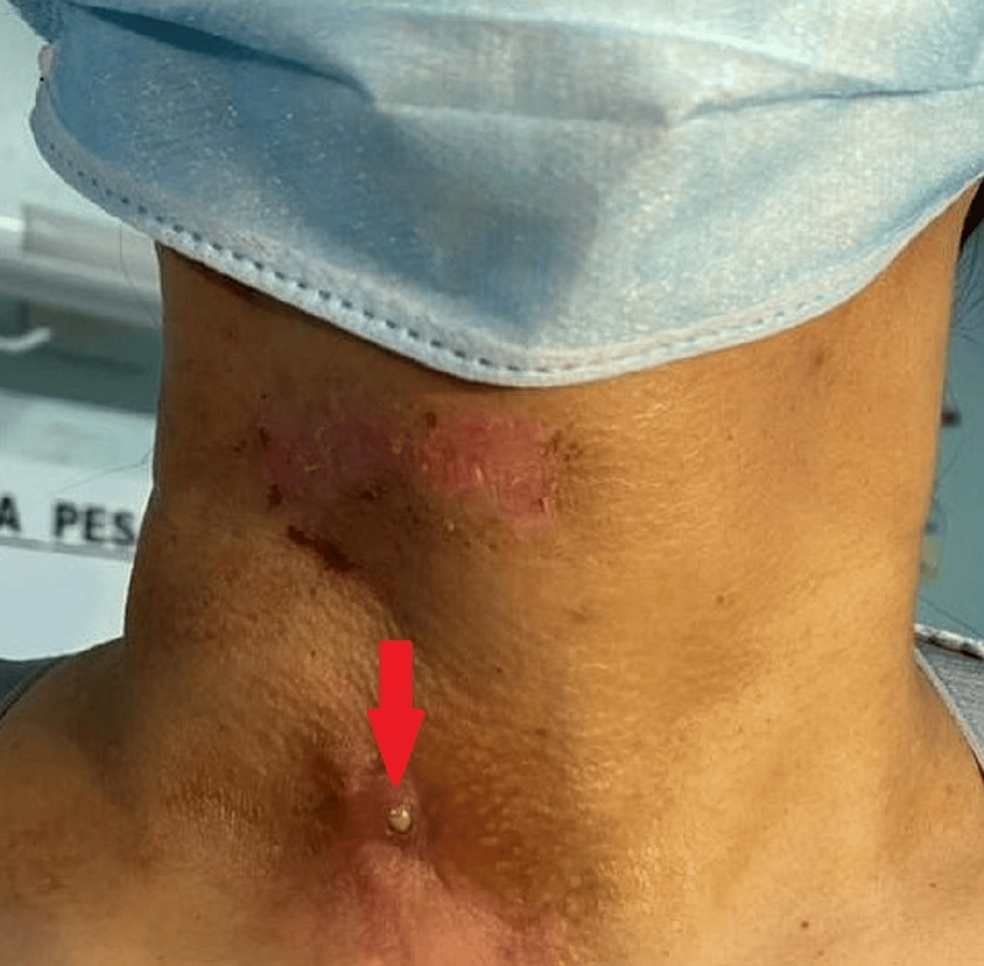
Patient’s neck with a small external opening (red arrow) with mucoid discharge - right lower anterior neck medial to the sternocleidomastoid muscle.

During her visit to our clinic, our examination revealed indurated skin edges with a small punctum over the lower two-thirds of the right anterior neck, medial to the right sternocleidomastoid muscle (Figure 1). There was no palpable neck swelling. The punctum was then cannulated with blue branula (22 G) and injected with methylene blue. Simultaneously, the flexible nasopharyngolaryngoscopy (FNPLS) examination demonstrated methylene blue flowing into the right pyriform sinus with an opening seen at the medial aspect of the right pyriform sinus. She was initially planned for chemical cauterization of the right branchial fistula with an intralesional injection of trichloroacetic acid under local anesthesia in the subsequent week. However, the procedure was abandoned as the fistula tract was unable to be cannulated and was no longer patent or fibrosed.

**Figure 2:**
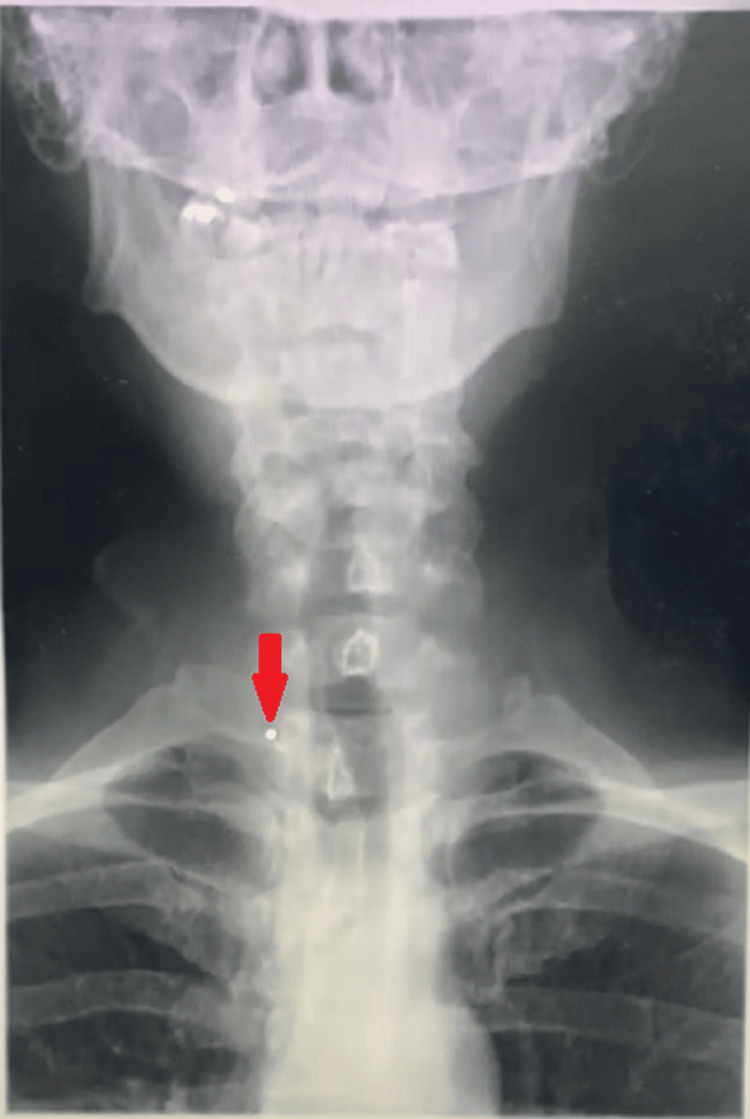
Barium swallow showing the retention of contrast at the right side of the neck (red arrow).

A month later, she presented with an abscess on her right neck which was resolved with incision and drainage as well as antibiotics. Her wound healed after two weeks without any more punctum or neck discharge. However, there were recurrent discharges six weeks later. The neck examination showed punctum with serous discharges over the previous site. The punctum again was successfully cannulated with blue branula (22 G) but with resistance felt upon a trial injection of normal saline. A barium swallow study followed by computed tomography (CT) scan showed a fistulous tract from the right pyriform sinus to the skin surface at the right lower anterior neck, coursing through the right upper pole of the thyroid gland and anterior to the right sternocleidomastoid muscle (Figure 2).

**Figure 3:**
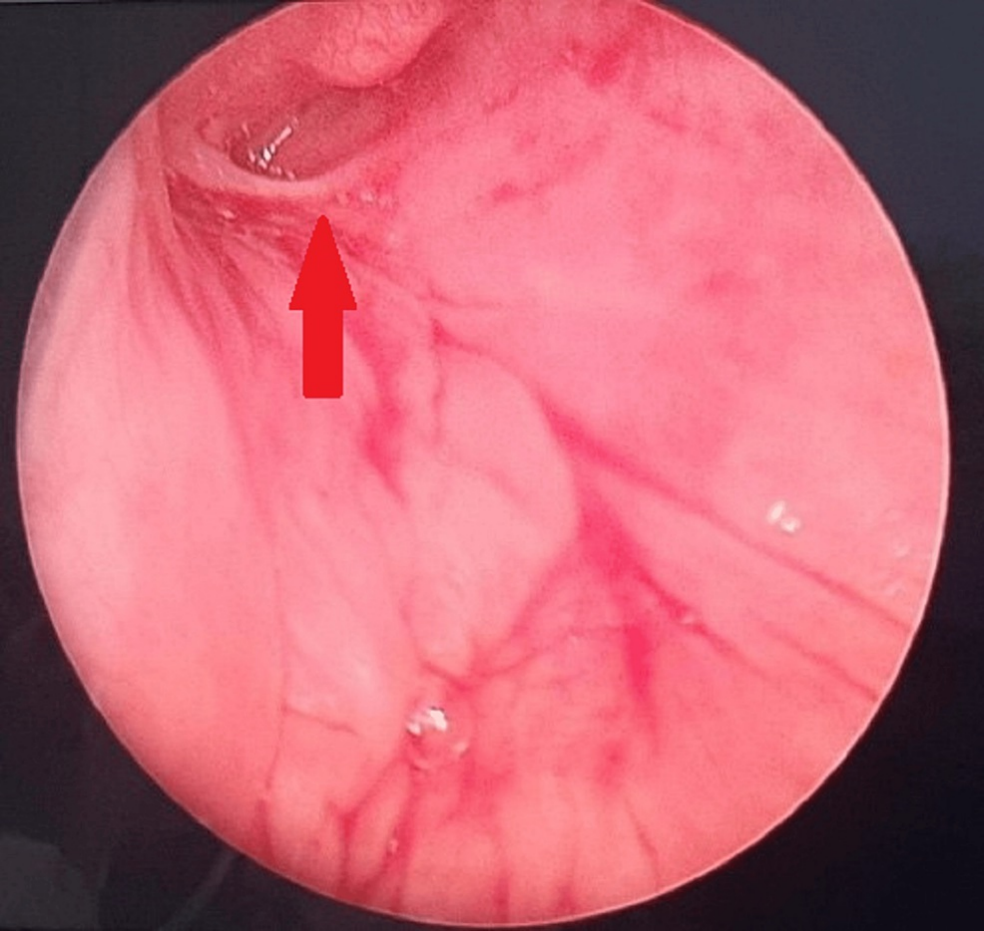
Direct laryngoscope showing the internal opening at the apex of the right pyriform sinus (red arrow).

**Figure 4:**
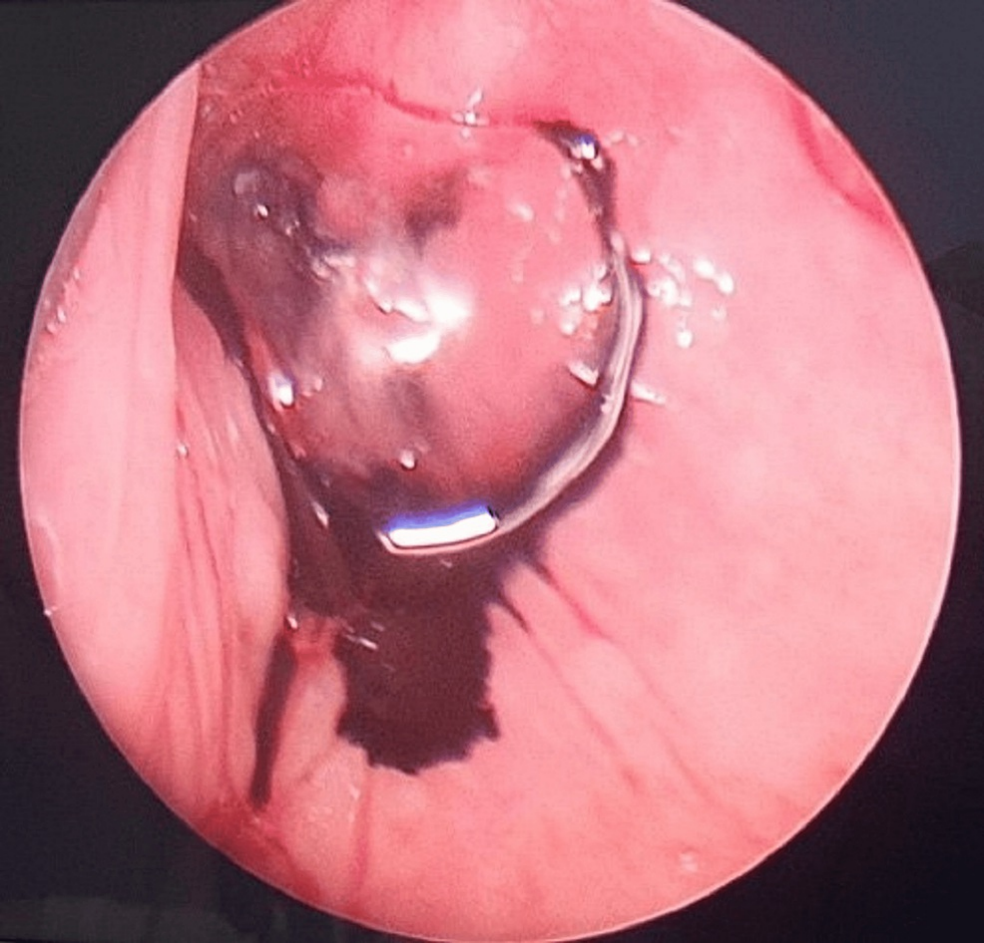
Extravasation of dye visualized from the internal opening.

**Figure 5:**
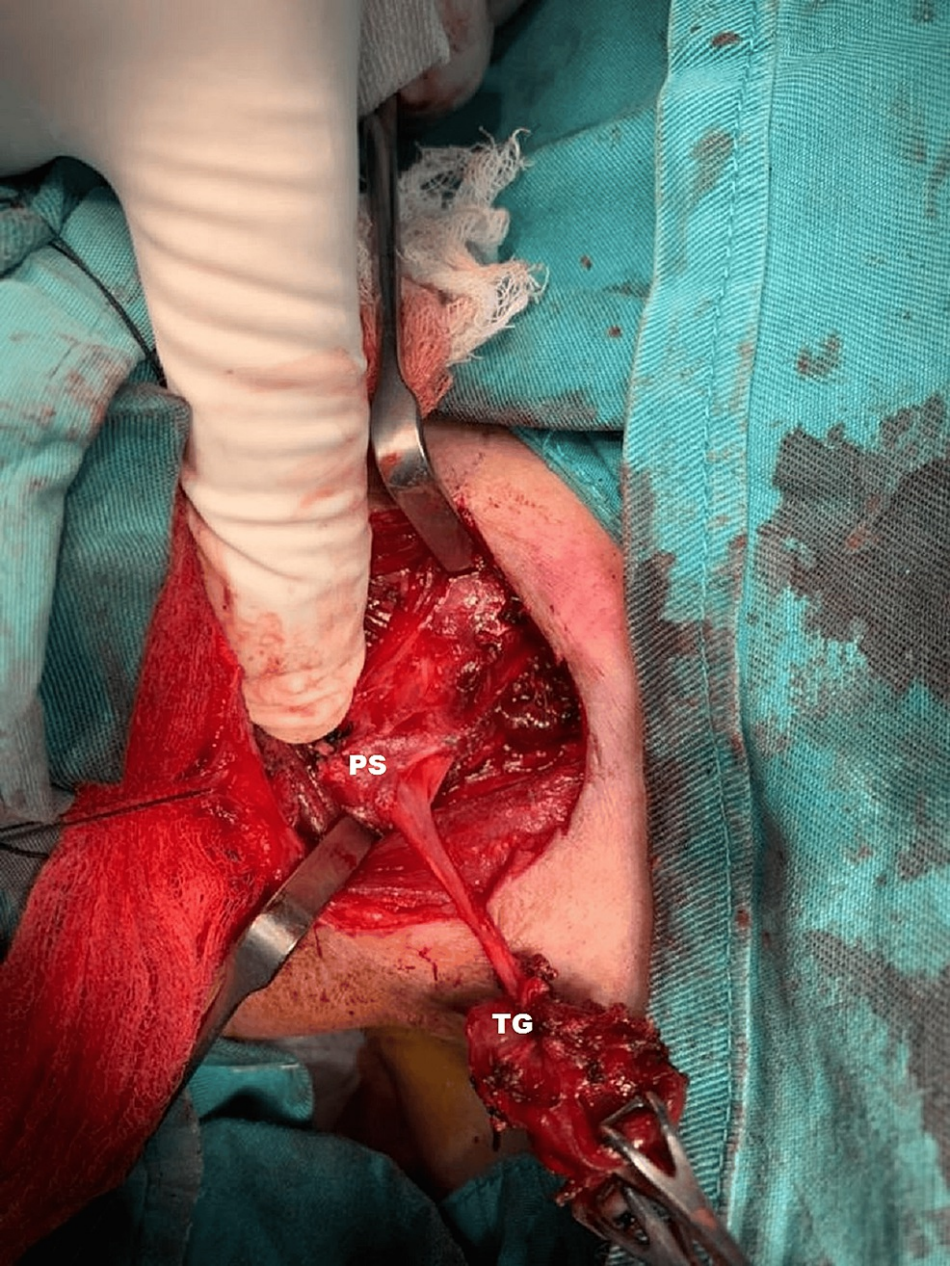
Intraoperative excision of the fistula tract from the superior pole of the right thyroid gland (TG) extending into the right pyriform sinus (PS).

**Figure 6:**
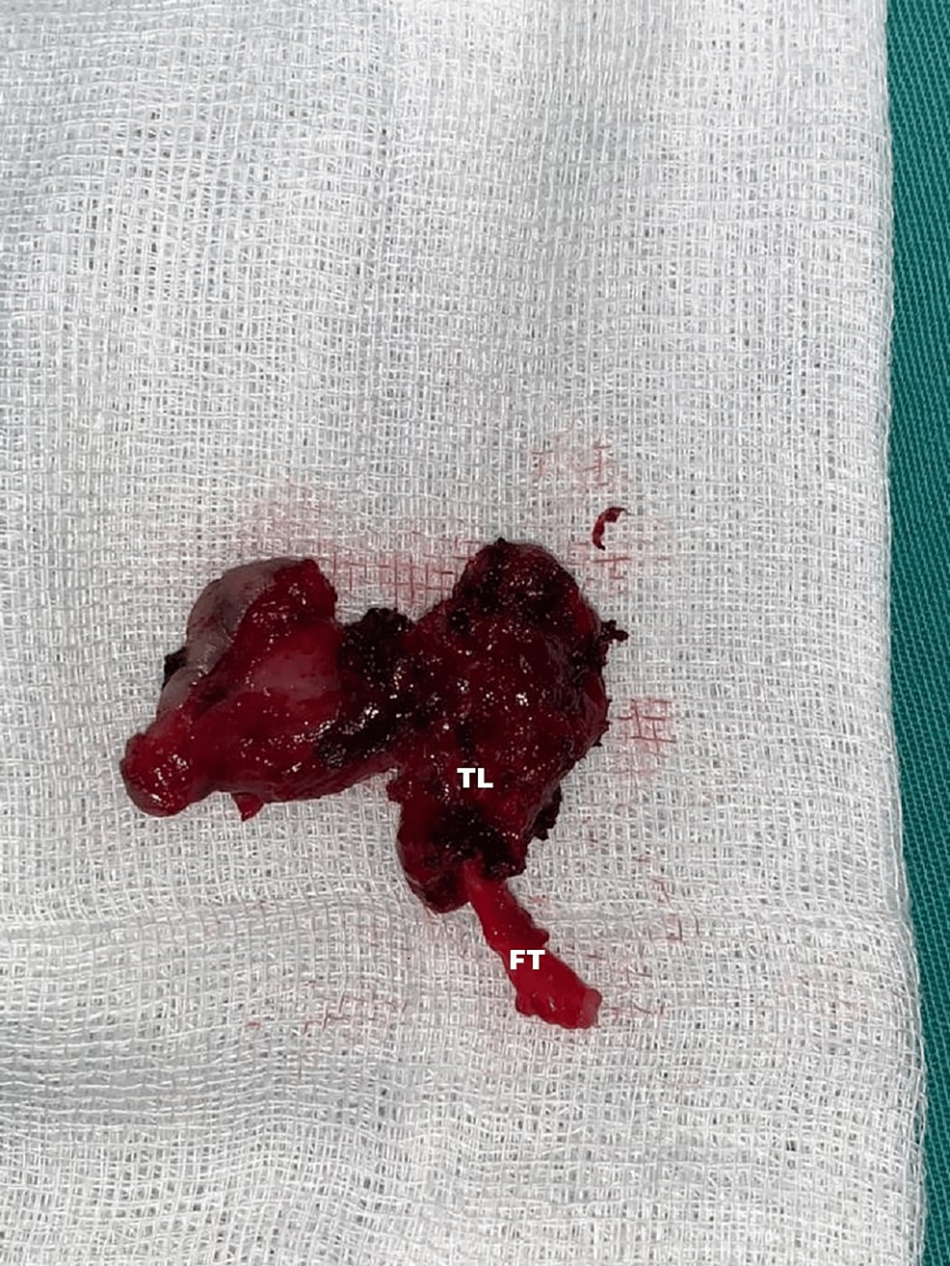
Fistula tract (FT) removed completely together with the right thyroid lobe (TL).

**Figure 7:**
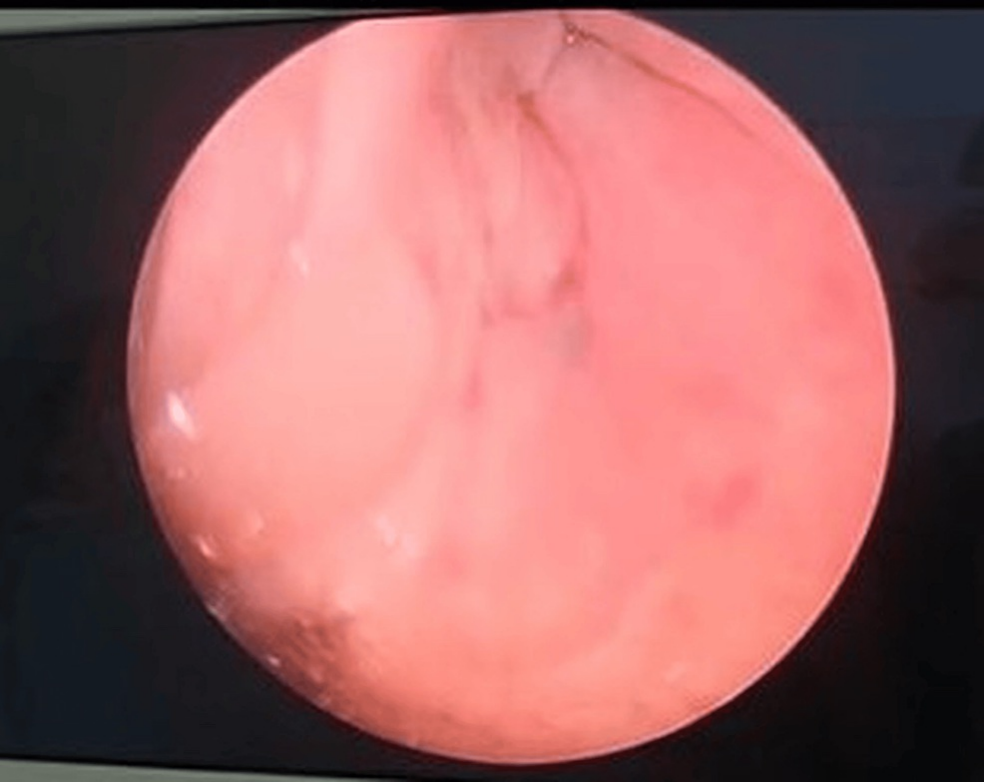
Previously seen internal opening at the right pyriform fossa no longer visualized after the operation.

Finally, complete surgical excision of the right branchial fistula was performed under general anesthesia. Intraoperatively, direct laryngoscopy showed an internal opening of the fistula at the apex of the right pyriform fossa (Figure 3). The external opening of the right branchial fistula was then cannulated with green branula sized 18 G and about 0.5 cc of methylene blue was injected with extravasation of dye visualized from the internal opening (Figure 4). Subsequently, excision of the right branchial fistula tract was performed. An elliptical incision around the external opening was made and the surrounding fibrotic tissues were removed together with the tract which involved platysma, parts of strap muscles, and the superior pole of the right thyroid gland until the internal opening into the right pyriform sinus (Figure 5). The tract was completely excised with a right thyroid lobectomy (Figure 6). The right recurrent laryngeal nerve and superior and inferior parathyroid glands were identified and preserved. The pyriform sinus defect was repaired in two layers with vicryl 4/0 (mucosa layer and constrictor muscle) followed by the opposition of strap muscles. Direct laryngoscopy was repeated at the end of the surgery, and the internal opening was no longer visualized (Figure 7).

Postoperatively, she was initially put on enteral feeding via a nasogastric tube which was removed on day 10 post-operation after normal FNPLS and barium swallow study. She was well after six months of operation with no recurrent neck discharges.

## Discussion

Fourth branchial anomalies typically manifest in the first decade of life and usually have a long history of recurrent neck lesions which are refractory to treatment [9]. Stadler et al. reported a case of recurrent neck infections with long-term misdiagnosis for 24 years before the correct diagnosis of fourth branchial cleft anomaly was made [10]. The correct diagnosis is crucial in providing the right management and preventing recurrent neck infections which could be fatal. Tong et al. reported a fatal case of severe neck abscess involving a third branchial fistula [11].

In our case, the patient presented to our clinic at the age of 46 years with a history of recurrent right anterior neck discharges and infections since childhood, which did not resolve despite multiple courses of antibiotics and surgical drainage of abscesses. There was a dilemma in diagnosis as the initial barium swallow was normal and the fistulogram only showed a short sinus. The challenge in diagnosis was because of the tract fibrosis and surrounding edema with recurrent infections. The diagnosis of branchial fistula could have been missed if there were no attempts to cannulate the fistula tract and injection of methylene blue as well as utilizing the endoscopic view of extravasation of dye through the internal opening at the right pyriform sinus.

The fistulous tract between the fourth and third branchial fistula can be differentiated by their relationships to the superior laryngeal nerve. The fourth branchial fistula exits the pharynx by passing inferior to the superior laryngeal nerve while the third branchial fistula exits the pharynx superior to the superior laryngeal nerve with the internal opening of the third fistula usually located higher up in the lateral wall rather than at the apex of pyriform fossa [12]. In our case, the internal opening was seen at the apex of the pyriform sinus, thus the diagnosis of fourth branchial fistula was made.

Various imaging modalities are available to delineate the type and extent of the fistulous tract, including the barium swallow study, fistulogram, ultrasound, CT scan, and magnetic resonance imaging. Hosokawa et al. reported that barium esophagography after resolution of the acute infection had a perfect 100% positive predictive value (PPV) in diagnosing branchial fistula, while other methods such as CT scan with oral contrast had a PPV of 88.9%, intravenous contrast-enhanced CT scan had a PPV of 53.8%, CT scan without contrast had a PPV of 33.3%, and ultrasound examination has the lowest PPV of 7.9% [13]. The fistula tract may become narrow in response to edema during the inflammatory period which prevents the passage of barium; hence, the barium study should practically be done after the resolution of acute infection. CT and MRI are useful for preoperative anatomical study for surgical planning.

As treatment of these disorders with repeated incision and drainage offers high rates of recurrence, complete excision of the fistulous tract during a quiescent period appears preferable [14]. Treatment should not be delayed once the diagnosis has been established as this can minimize the usage of antibiotics to treat acute infections and prevent antibiotic resistance in individuals. A more conservative approach by endoscopic cauterization of the internal opening can be done using trichloroacetic acid, silver nitrate, plasma, CO_2_ laser, or electrocautery. Although this method is relatively safer compared to open neck surgery, it does have a risk of recurrent laryngeal nerve or even esophageal injury with a significantly increased risk of recurrence [8].

Surgical excision of the branchial fistula can either be an external excision of the tract with partial thyroidectomy plus endoscopic cauterization of internal opening or complete excision of the tract up to the pyriform fossa. The surrounding fibrotic tissues and involved thyroid tissue should be resected en bloc to prevent recurrence [6]. In our case, complete excision of the right branchial fistula tract was done with removal of the surrounding fibrotic tissues, thyroid lobectomy followed by repair of defect at the pyriform fossa. There was no evidence of recurrence after six months of operation.

## Conclusions

Fourth brachial fistulas are rarely seen. It is crucial to obtain the correct diagnosis for prompt treatment. Recurrent neck infections or abscesses should raise a high clinical suspicion of possible branchial anomaly and should be investigated thoroughly. Barium swallow after resolution of acute infection and endoscopy are important tools for diagnosis. Complete excision of the fistulous tract is the mainstay of treatment to prevent the recurrence of disease and provide a better outcome for the patient.
